# Patients’ perceptions of service quality in China: An investigation using the SERVQUAL model

**DOI:** 10.1371/journal.pone.0190123

**Published:** 2017-12-22

**Authors:** Li-hua Fan, Lei Gao, Xin Liu, Shi-hong Zhao, Hui-tong Mu, Zhe Li, Lei Shi, Ling-ling Wang, Xiao-li Jia, Min Ha, Feng-ge Lou

**Affiliations:** 1 Department of Health Management, School of Public Health, Harbin Medical University, Harbin, Heilongjiang, People’s Republic of China; 2 Department of Neurosurgery, The Second Affiliated Hospital of Harbin Medical University, Harbin, Heilongjiang, People’s Republic of China; 3 Department of Autonomous Protection, Chinese Hospital Association, Beijing, People’s Republic of China; 4 Department of Public Health Research, School of Public Health, Qiqihar Medical University, Qiqihar, Heilongjiang, People’s Republic of China; TNO, NETHERLANDS

## Abstract

**Background and aim:**

The doctor–patient relationship has been a major focus of society. Hospitals’ efforts to improve the quality of their medical services have been to reduce the probability of doctor–patient conflicts. In this study, we aimed to determine the gap between expectations and perceptions of service quality according to patients to provide reference data for creating strategies to improve health care quality.

**Methods:**

Twenty–seven hospitals in 15 provinces (municipalities directly beneath the central government) were selected for our survey; we sent out 1,589 questionnaires, of which 1,520 were collected (response rate 95.65%) and 1,303 were valid (85.72% effective recovery rate). Paired t-tests were used to analyze whether there were significant differences between patients' expectations and perceived service quality. A binary logistic regression analysis was used to determine whether there were significant differences in the gap between expectation and perception of service quality according to patients' demographic characteristics.

**Results:**

There was a significant difference between the expected and perceived service quality (p < 0.05) according to patients both before and after receiving medical services. Furthermore, the service quality gap of each service dimension was negative. Specifically, the gaps in service quality were as follows: economy, responsiveness, empathy, assurance, reliability, and tangibles. Overall, we can conclude that patients’ perceptions of service quality are lower than their expectations.

**Conclusions:**

According to the study results, the quality of health care services as perceived by patients was lower than expected. Hospitals should make adjustments according to the actual situation and should strive to constantly improve the quality of medical services for patients.

## Introduction

Recently, with the improvement of people’s living standards, customers are becoming increasingly attentive to obtaining the best-quality products. Accordingly, in the medical field, patients are paying increasing attention to the quality of medical services. Understanding the quality of their medical services can help organizations identify their own competitive advantages and disadvantages while, at the same time, preventing waste of resources [1]. Medical service quality has been found to be associated with patient satisfaction [2]. When patients experience satisfactory medical treatment, their trust in the hospital tends to increase, which, in turn, benefits the construction of harmonious doctor–patient relationships [3]. Therefore, accurately understanding the needs and expectations of patients regarding medical services as well as the gap in patients’ expectations and perceptions of service quality is exceedingly important for improving the quality of hospital care services.

The concept of customer service quality was initially proposed in the early 1980s. In 1982, a professor in Finland—Christian Gronroos [4]—proposed the concept of the customers’ perceived service quality and created the perceived service quality model. He interpreted service quality as a subjective construct that depended on contrasting customers’ expectations of the quality of a service (i.e., the expected service quality) with their perceptions of the actual quality of the service (perceived service quality).

In the mid-1980s, Parasuramn A, Valarie A. Zeithaml, and Leonard L. Berry (PZB) [5] began to study factors related to customers’ perceptions and decisions regarding service quality. In 1985, the three of them published an article titled “A conceptual model of service quality and its implication for future research” in the *Journal of Marketing*, in which they put forward the “service quality gap model.” While this model originally had 10 dimensions, they cut it down to five—tangibles, reliability, responsiveness, assurance, and empathy—which are described as follows:

1. Tangibles: physical facilities, equipment, and appearance of personnel
2. Reliability: ability to perform the promised service dependably and accurately
3. Responsiveness: willingness to help customers and provide prompt service
4. Assurance (including competence, courtesy, credibility, and security): knowledge and courtesy of employees and their ability to inspire trust and confidence
5. Empathy (including access, communication, and understanding the customer): caring and individualized attention that the firm provides to its customers

Next, PZB [6] developed a service quality evaluation instrument they termed “SERVQUAL.” After numerous modifications, SERVQUAL comprised 22 items in five subscales, which corresponded to the dimensions of the perceived service quality gap model [7]. Since its development, SERVQUAL has been used in numerous service sectors, including telecoms, health care, fast food, enterprise, banking, tourism, and higher education. SERVQUAL is also widely used in the medical field [8].

Indeed, numerous scholars have used SERVQUAL to evaluate medical service quality [9–12]. For instance, Teng et al. [9] used SERVQUAL to assess patients in surgical departments and confirmed that the instrument was valid and reliable in this population. In China, several scholars have examined patients’ perceptions of service quality. In 2004, Niu Hongli introduced the SERVQUAL evaluation system to the medical field in China. Based on SERVQUAL, they created an index system for a medical service quality evaluation scale and studied the optimal method of reading the scale. Many studies describe the application of SERVQUAL in the evaluation of medical service quality [13–17]. Yang Jia et al. [15] surveyed 216 outpatients in a hospital in Beijing and found a large service quality gap overall; by dimension, the tangibility dimension had the smallest gap.

According to the above mentioned studies, Chinese patients appear to be generally dissatisfied with the quality of medical services. However, it is notable that the medical service industry in China is special; thus, it would be necessary to adjust the items and dimensions of SERVQUAL to fit the special characteristics of China's medical industry [18–19]. Past studies have shown that SERVQUAL can feasibly be used to evaluate China's medical services. Previous researchers [6–10] have mainly focused on patients from a single hospital in a limited region; thus, the scope of investigation is limited. In this study, we surveyed 27 hospitals in 15 provinces (i.e., municipalities directly under the central government). This ensures that the sample size was large and the coverage wide. We aimed to compare patients’ expectations of service quality and their perceptions of the quality of services actually received and explored the factors underlying the differences in perception. To meet the demands of patients, most hospitals should improve the quality of their services.

## Materials and methods

### Sample design and data collection

Data were collected between January and June of 2016. The sample was selected using convenience sampling. In 27 hospitals across 15 provinces in China, we administered questionnaires to 1,589 hospitalized patients or their relatives (hospitalized for more than three days) who were over 18 years of age and had the capacity for independent judgment. Specifically, the participating hospitals are shown in Table 1. The researchers entered the inpatient ward under the condition of seeking hospital approval and obtained the informed consent of the patients or their families. The researchers issued the questionnaires at the scene, and they were completed by the patients or their families. The investigators recovered questionnaires on the spot. We sent out 1,589 questionnaires, of which 1,520 were collected (response rate 95.65%) and 1,303 were valid (85.72% effective recovery rate).

**Table 1:** The participant hospital name.

Number	Hospital name	Location
1	Beijing Tian Tan Hospital, Capital Medical University	Beijing
2	Beijing Ji Shui Tan Hospital	Beijing
3	General Hospital of Chinese people's Armed Police Force	Beijing
4	Chinese Pla General Hospital	Beijing
5	Anhui No 2. Province People’s Hospital	Anhui Province
6	The First People's Hospital of Hefei	Anhui Province
7	Anhui Provincial Hospital	Anhui Province
8	Jinan Central Hospital	Shandong Province
9	Zibo Central Hospital	Shandong Province
10	Liaocheng People's Hospital	Shandong Province
11	Henan Province People's Hospital	Henan Province
12	The Second Hospital of Shanxi Medical University	Shanxi Province
13	The First Hospital of China Medical University	Liaoning Province
14	Taihe Hospital	Hubei Province
15	Shaanxi Provincial Armed Police Corps Hospital	Shaanxi Province
16	Shaanxi Provincial People’s Hospital	Shaanxi Province
17	The Second Affiliated Hospital Of Xi’an Jiaotong University(Xibei Hospital)	Shaanxi Province
18	Gansu Second People's Hospital	Gansu Province
19	The First Hospital Of Lanzhou University	Gansu Province
20	Affiliated Hospital of Tianjin Army Logistics College	Tianjin
21	Peiking University Binhai Hospital	Tianjin
22	Hebei General Hospital	Hebei Province
23	The First Hospital of Shijiazhuang	Hebei Province
24	Nanfang Hospital	Guangdong Province
25	Xiangya Hospital Central South University	Hunan Province
26	General Hospital Of Ningxia Medical University	Ningxia
27	The Second Affiliated Hospital of Harbin Medical University	Heilongjiang Province

### Design and development of questionnaire

The questionnaire was designed using the following steps. First, we referred to the international standards for SERVQUAL [6] and the actual situation of the medical service sector in China to make appropriate changes to form our questionnaire. Next, we carried out a preliminary investigation in three hospitals in Harbin. In this preliminary survey, we issued 75 questionnaires; these 75 individuals were not included in the formal study. After processing the preliminary data, we further modified the questionnaire. Finally, we consulted health management experts, hospital administrators, clinicians, and other health experts (a total of six persons) for expert opinions on the questionnaire to further improve the formation.

Through the literature review, pre-investigation, and expert consultation, the final questionnaire was formed. This questionnaire comprised general characteristics (age, sex, education, income, clinic department, medical payments) and a 24-item scale each for expectations and perceptions [20–22]. (Patient expectation is the expected health service before receiving medical services. It is influenced by past experience, public opinion, the image of medical institutions, and oral communication from relatives and friends. Patient perception refers to the patient's actual feelings of the quality of service provided by the hospital after receiving medical service.) The scale comprised the following dimensions: tangibles (items 1–5), reliability (items 6–9), responsiveness (items 10–13), assurance (items 14–18), empathy (items 19–21), and economy (items 22–24), all rated on a 5-point Likert scale (strongly disagree, disagree, indifferent, agree, and strongly agree). Higher scores on each item indicate that patients’ expectations and perceptions regarding the quality of medical services are more positive.

The results of validity testing indicated that all dimensions met the minimum validity requirements. Regarding the reliability, the Cronbach’s alpha value for expectations of service quality was 0.967 for the whole scale; those for the six dimensions all exceeded 0.8. For perception of service quality, the Cronbach’s alpha of the whole scale was 0.933, and those for the six dimensions were all over 0.7. The details are shown in Table 2.

**Table 2:** Analysis of the reliability of the service quality questionnaire.

Dimensions	Cronbach’s Alpha	
	Expectations	Perceptions
Tangibles	0.882	0.816
Reliability	0.891	0.829
Responsiveness	0.905	0.848
Assurance	0.927	0.871
Empathy	0.895	0.796
Economic	0.898	0.879
Total	0.967	0.933

The principal component analysis method was used to extract the factor with the characteristic value greater than 1 and the factor load greater than 0.45 according to the Kaiser standard. The results show that expectations and perception of the Kaiser-Meyer-Olkin values were 0.965 and 0.933, more than 0.7. Bartlett sphericity test values were 28108.413 and 18984.452. Degrees of freedom was 276. P values reached a significant level (<0.001), indicating good questionnaire structure validity.

### Data calculation method

The difference between perceptions (P) and expectations (E) (P − E = SQ) represents service quality. When SQ is negative, there is a service quality gap. Conversely, when SQ is positive, patients' expectations are greater than their perceptions [23]. Each dimension of the specific calculation is shown in Table 3.

**Table 3:** Main calculation method.

Service dimensions	Calculating SERVQUAL scores
SQ1=Tangibles	SQ1=[(P1-E1)+(P2-E2)+(P3-E3)+(P4-E4)+(P5-E5)]/5
SQ2=Reliability	SQ2=[(P6-E6)+(P7-E7)+(P8-E8)+(P9-E9)]/4
SQ3=Responsiveness	SQ3=[(P10-E10)+(P11-E11)+(P12-E12)+(P13-E13)]/4
SQ4=Assurance	SQ4=[(P14-E14)+(P15-E15)+(P16-E16)+(P17-E17)+(P18-E18)]/5
SQ5=Empathy	SQ5=[(P19-E19)+(P20-E20)+(P21-E21)]/3
SQ6=Economic	SQ6=[(P22-E22)+(P23-E23)+(P24-E24)]/3

### Data analysis method

Initially, we performed data entry using Epidata and then employed SPSS Statistics 20 for the statistical analysis. We calculated descriptive statistics (means and standard deviations) for patients’ expectations and perceptions of service quality. Paired-sample t-tests were used to compare the expectations and perceptions of service quality and to determine which services have the greatest gaps in quality. When p < 0.05, the results were statistically significant. A binary logistic regression analysis was used to examine the relationship between patients’ and their families’ expected and perceived gaps of service quality and demographic characteristics.

### Ethical approval

This research project was approved by the Medical Ethics Committee of the School of Public Health, Harbin Medical University. Before the survey, we received approval from the research hospitals; furthermore, all participants participated voluntarily and anonymously after signing informed consent forms. The collected data did not contain personal information such as name, telephone, and so on, so they are completely confidential.

## Results

### Patient characteristics

According to the survey results, there was a relatively equal proportion of male (47.8%) and female (52.2%) participants. Most participants were treated in the internal medicine (40.1%) and surgery (24.5%) departments, and their main methods of paying for their medical services were basic medical insurance for urban workers (25.6%), basic medical insurance for urban residents (25.6), and the new rural cooperative medical system (27.7%). Most (96.6%) patients were aware of their illnesses while 90.9% were aware of the treatment of their diseases; 50.1% of patients felt satisfied with their doctors. The specific characteristics are shown in Table 4.

**Table 4:** Descriptive statistics of patients’ basic characteristics.

**A**			
**Variable**	**Category**	**N**	**%**
Gender			
	Female	680	52.2
	Male	623	47.8
Age (years)			
	31-40	364	27.9
	18-30	320	24.6
	41-50	240	18.4
	>60	217	16.7
	51-60	162	12.4
Education levels			
	High school	458	35.1
	Undergraduate	449	34.5
	Junior and below	359	27.6
	Postgraduate	37	2.8
Average income per month (yuan)			
	1001-3000	501	38.4
	3001-5000	385	29.5
	5001-8000	188	14.4
	<1000	119	9.1
	> 8001	110	8.4
The medical department			
	Internal medicine	534	41.0
	Surgery	319	24.5
	Paediatrics	230	17.7
	Else	130	10.0
	Gynaecology and obstetrics	60	4.6
	Ophthalmology and otorhinolaryngology	14	1.1
	Department of stomatology	11	0.8
	Image inspection section	5	0.4
The main payment method			
	The new rural cooperative Medical care	361	27.7
	Urban workers basic medical Insurance	333	25.6
	Urban residents' basic medical Insurance	333	25.6
	Entirely at his own expense	231	17.7
	Commercial insurance	37	2.8
	Else	8	0.6
**B**			
The disease is clear			
	Yes	1259	96.6
	No	44	3.4
Treatment is clear			
	Yes	1184	90.9
	No	119	9.1
The doctors are satisfied			
	Satisfied	653	50.1
	Very Satisfied	459	35.2
	General	169	13.0
	Dissatisfied	17	1.3
	Very dissatisfied	5	0.4

### Gaps between expectation and perception of service quality according to patients’ demographic characteristics

Survey objects differed according to clinic departments, which is the exposure factor of the tangibility service quality gap. Among these, the gap of tangible service quality in the gynecological survey objects was 2.367 times that of other departments (OR = 2.367, 95% CI 1.243 to 4.505). The gap of responsiveness service quality in the male participants was 0.690 times that of female participants (OR = 0.690, 95% CI 0.553 to 0.860). The gap in the assurance service quality in the male participants was 0.760 times that of female participants (OR = 0.760, 95% CI 0.607 to 0.952). The results are shown in Table 5 and Table 6.

**Table 5:** Associations between patients' demographic characteristics and the mean gaps of the tangibilityand empathy dimensions of service quality: Binary logistic results.

Characteristics	Dimensions					
		Tangibility			Empathy	
	OR	(95%C.I.)	P-value	OR	(95%C.I.)	P-value
**Sex**						
Male				1.237	0.640–0.992	0.282
Female				1	Reference	
**Income Levels (yuan)**						
<1000				0.871	0.518–1.465	0.603
1001-3000				0.885	0.585–1.338	0.562
3001-5000				1.134	0.741–1.736	0.561
5001-8000				1.358	0.844–2.185	0.207
>8000				1	Reference	
**The medical department**						
Internal medicine	1.364	0.929–2.003	0.113			
Surgery	1.118	0.743–1.681	0.593			
Gynaecology and obstetrics	2.367	1.243–4.505	0.009			
Paediatrics	1.616	1.047–2.492	0.030			
Department of stomatology	1.919	0.536–6.874	0.317			
Ophthalmology and otorhinolaryngology	1.462	0.480–4.451	0.503			
Image inspection section	0.731	0.118–4.522	0.736			
Else	1	Reference				

OR: Odds ratios, CI: Condence interval, Reference: reference category in the logistic regression model

**Table 6:** Associations between patients' demographic characteristics and the mean gaps of the reliability,responsiveness,assurance and economic dimensions of service quality: Binary logistic results.

Characteristics	Dimensions											
	Reliability			Responsiveness			Assurance			Economic		
	OR	(95%C.I.)	P-value	OR	(95%C.I.)	P-value	OR	(95%C.I.)	P-value	OR	(95%C.I.)	P-value
**Sex**												
Male				0.690	0.553–0.860	0.001	0.760	0.607–0.952	0.017			
Female				1	Reference		1	Reference				
**Age (years)**												
18-30							1.250	0.866–1.804	0.234	1.042	0.725–1.496	0.826
31-40							1.068	0.750–1.520	0.716	0.869	0.610–1.237	0.435
41-50							0.876	0.603–1.273	0.488	0.592	0.406–0.863	0.006
51-60							0.739	0.490–1.114	0.148	0.869	0.577–1.310	0.503
>61							1	Reference		1	Reference	
**Education Levels**												
Junior and below	0.801	0.399–1.608	0.533	0.787	0.394–1.570	0.496				0.381	0.182–0.796	0.010
High school	0.588	0.295–1.171	0.131	0.592	0.298–1.175	0.134				0.407	0.198–0.838	0.015
Undergraduate	0.738	0.370–1.470	0.387	0.820	0.413–1.627	0.571				0.559	0.273–1.147	0.113
Postgraduate	1	Reference		1	Reference					1	Reference	

OR: Odds ratios, CI: Condence interval, Reference: reference category in the logistic regression model

### Mean service quality gaps by item

According to the survey, aside from item 3 (“hospital medical staff wear clean and decent uniforms”), the remaining items showed negative service quality gaps; the differences between expectations and perceptions were significant (p < 0.05). This information indicates that patients’ expectations were not met. The greatest gap was for item 22 (“the hospital medical expenses are reasonable”) followed by item 23 (“the cost of medical services is issued in a timely and convenient manner”) and then item 24 (“detailed list of the items in the hospital charges”). As shown in Table 7, a total of 24 items showed a significant gap (p < 0.05); it also shows the difference in expectation and perception before and after receiving medical care.

**Table 7:** Mean service quality gaps by item.

Number	Expectation	Perception	Service gap	t-value	P-value
1	4.01	3.92	-0.09	4.078	<0.001
2	3.90	3.77	-0.13	4.415	<0.001
3	3.97	4.08	0.11	-4.131	<0.001
4	3.96	3.85	-0.11	3.735	<0.001
5	3.93	3.85	-0.08	3.048	0.002
6	4.09	3.97	-0.12	4.622	<0.001
7	4.12	4.05	-0.07	2.531	0.012
8	4.12	3.94	-0.18	6.677	<0.001
9	4.18	4.05	-0.13	4.911	<0.001
10	4.18	3.95	-0.23	7.986	<0.001
11	4.19	3.90	-0.29	9.982	<0.001
12	4.11	3.80	-0.31	9.874	<0.001
13	4.15	3.83	-0.32	9.596	<0.001
14	4.14	3.95	-0.19	5.866	<0.001
15	4.20	4.06	-0.14	5.208	<0.001
16	4.23	4.07	-0.16	6.121	<0.001
17	4.25	4.10	-0.15	5.734	<0.001
18	4.30	4.09	-0.21	8.012	<0.001
19	4.25	4.02	-0.23	8.314	<0.001
20	4.15	3.98	-0.17	6.127	<0.001
21	4.09	3.72	-0.37	11.757	<0.001
22	3.89	3.44	-0.45	13.708	<0.001
23	4.01	3.57	-0.44	12.956	<0.001
24	4.03	3.61	-0.42	12.190	<0.001

Please see questionnaire about 1–24 items’ specific content.

### Patients’ expectations and perceptions of the quality of provided services

This study calculated the service quality gap according to dimension of service quality. The results showed that patients had the highest expectations for the assurance dimension (mean 4.224), followed by empathy, responsiveness, reliability, economy, and tangibles. Regarding the perceived quality of the services, assurance was again highest, followed by reliability, empathy, tangibles, responsiveness, and economy. Regarding the service quality gaps, the greatest was for economy, followed by responsiveness, empathy, assurance, reliability, and tangibles. We observed significant differences in patients’ expectations and perceptions before and after receiving medical services (p < 0.05). See Table 8 for details.

**Table 8:** Patients’ expectations and perceptions of the quality of provided services.

Dimensions	Expectations	Perceptions	Gap scores	P-value	Rank
Tangibles	3.9540	3.8940	-0.0600	0.003	6
Reliability	4.1275	4.0025	-0.1250	<0.001	5
Responsiveness	4.1575	3.8700	-0.2875	<0.001	2
Assurance	4.2240	4.0540	-0.1700	<0.001	4
Empathy	4.1630	3.9100	-0.2530	<0.001	3
Economic	3.9770	3.5400	-0.4370	<0.001	1
Total	4.2672	3.8784	-0.3888		

## Discussion

From patients’ perspectives, it is of great importance to ensure high-quality hospital care services. We explored patients’ expectations and perceptions of hospital service quality to determine the gap in hospital service quality, thereby providing accurate reference data for improving medical services.

### Demographic characteristics and service quality gap

Gynecology mainly concerns female patients, whose minds are more delicate and more sensitive to the gap in tangibility services quality and other perception aspects. It is suggested to improve the infrastructure construction and provide more convenient service facilities for more departments such as gynecology and pediatrics—for example, installation of toilet handrails for patients, rental seats for accompanying personnel, kettles, and so on.

In demographic characteristics, gender is significant in response and guaranteed quality of service gap. This suggests that hospitals should provide more detailed services to patients and provide patients with enough security when providing services. At the same time, because women constitute a vulnerable group in society, and the body and mind are more vulnerable in the face of disease, medical staff should pay more attention to the needs of female patients.

### Patients’ expected service quality

Patients’ expectations of service quality were ranked as follows (high to low): assurance, empathy, responsiveness, reliability, economy, and tangibles. These results differ from PZB’s ranking [4], which was reliability, responsiveness, assurance, empathy, and tangibles. This is likely due to differences in political, economic, and cultural factors between countries and statistical methods used by researchers, all of which contribute to differences in the demand for medical services.

### Patients’ perceptive service quality

Regarding their perceptions of service quality, patients gave the lowest ratings to the economy dimension. This is in accordance with the results indicating that 38.4% of patients had an income between 1000 and 3000 RMB and that medical services were mainly paid for using the new rural cooperative medical system, suggesting that patients’ economic level was rather low. Most rural patients are more sensitive to economic factors and cannot often afford excessive medical costs. Furthermore, even though most people in China have medical insurance, they still must partially cover their own treatment costs. This has likely led to a sizeable economic burden for some patients. Note that all of the surveyed hospitals were tertiary hospitals; compared with other hospitals, tertiary hospitals are costly and have a substantial outpatient clientele. Furthermore, medical staff are often too busy to inform patients of cost details in a timely manner, thus leading to lower levels of economic awareness among patients [24].

### Patients’ expected and perceived quality of service gap

The results of this study show that, for all six dimensions of service quality, the perceived quality was lower than expected. More specifically, the gaps were as follows, ranging from largest to smallest: economy, responsiveness, empathy, assurance, reliability, and tangibility. In other words, the service quality gap was largest for the economy dimension. The greatest gap in economy was in item 22 (“the hospital medical expenses are reasonable"). This may relate to China's doctor–patient contradiction of “expensive to see a doctor” and the reality of the relevant issues [18]. In other words, this gap is perhaps caused by both the excessively high medical costs in the hospitals and the low income of respondents, which suggests that the cost of medical care is, indeed, an economic burden [25].

The gaps for responsiveness and empathy were ranked second and third, respectively. The gaps were largest for items 13 (“medical staff willingness to help patients”) and 21 (“the hospital gives priority to your benefits, not the benefits of medical staff”). One reason for this can be explained with Maslow’s Hierarchy of Needs [26]. This theory indicates that people’s physiological, security, and social needs must be incrementally met. With the development of society and concomitant improvement in people’s living standards, patients are becoming less satisfied with a hospital providing only treatment for disease; in other words, they are seeking higher-level services. Thus, the quality of medical services is no longer limited to the physical care provided to patients but also includes their psychological care. Additionally, patients in China tend to believe that hospitals are “for profit” and, thus, do not prioritize patient interests. Because the government in the field of medical investment is inadequate, whether public or private, it is only profitable to ensure normal operation of the hospital [27]. However, ethically, hospitals should prioritize the safety of patients, which suggests that hospital managers must find a balance between these two demands.

The gaps for the assurance and reliability dimensions were ranked fourth and fifth, respectively. According to Table 4, most patients or their families are aware of their own condition and treatment and are satisfied with the doctor's treatment. These results indicate that patients tend to trust medical services and find their experience in the hospital to be relatively satisfactory with regard to their initial expectations.

The lowest gap in service quality was for tangibles. This result is consistent with the results of Yu Yawei and Yu Liling [13]. The reason that this gap was smallest may be that the tangibles of care are not overly important to patients. Alternatively, we selected participants from three large hospitals, which tend to have adequate medical equipment and other resources. Thus, while it did not meet patients’ expectations, it was nevertheless the smallest gap of all dimensions.

### Limitations

First, this research adopted a method requiring patients to recall their own situation. Thus, the results give rise to bias. Owing to the convenient sampling method, the samples selected are less representative. Limited by manpower, material resources, and financial resources, the scope of hospitals and the number of patients are limited.

## Conclusions

According to the results of this study, all six dimensions of service quality showed a negative gap, indicating that patients’ expectations were not met. The largest gap was for the economy dimension followed by responsiveness, empathy, assurance, reliability, and tangibility. This reflects the high cost of treating illnesses, which remains a major problem requiring urgent solution in China. Hospitals should make adjustments according to the actual situation of service quality and should constantly strive to improve the quality of medical services.

## Supporting information

S1 Dataset Supporting dataset. The supporting dataset includes the data underlying our findings in this study.
(XLS)

S1 Questionnaire (DOC)
