# Supplementary material for: Patients’ perceptions of service quality in China: An investigation using the SERVQUAL model
Source: PLoS One. 2017 Dec 22;12(12):e0190123. doi: 10.1371/journal.pone.0190123 (PMC5741236; doi:10.1371/journal.pone.0190123)
Supplement: S1 Questionnaire — (DOC) [file pone.0190123.s002.doc]

**Questionnaire on patient expectations and perceptions of medical services**

**Questionnaire number**：____________

Dear ladies and gentlemen：

Hello！We will understand your on inpatient service quality expectations and actual experience of the situation through the investigation, to analyze the existing in the hospital inpatient service quality problems, to further improve the quality of hospital inpatient services provide opinions and suggestions.The information of this questionnaire is only used in this subject,using anonymous way to answer.！Your information will be kept strictly confidential. Please fill the question in your free time. Thank you for your support

**The first part of the basic situation**

1.Age：Male□ Female□

2.Age： 18-30 years□ 31-40 years□ 41-50 years□ 51-60 years□ above 61 years□

3.Education level:Junior and below□ High school□ Undergraduate□ Postgraduate□

4.Average income per month ：Less than1000 yuan□ 1001-3000 yuan□ 3001-5000 yuan□ 5001-8000 yuan□ Above 8001 yuan□

5.The medical department：Internal medicine □ Surgery□ Gynaecology and obstetrics□ Paediatrics□ Department of stomatology□ Ophthalmology and otorhinolaryngology□ Image inspection section□ Else□

6.The main payment method:

Entirely at his own expense□ Urban workers basic medical insurance □Urban residents' basic medical insurance□ The new rural cooperative medical care□ Commercial insurance □Else□

7.Are you aware of the disease in your hospital? Yes□ No□

8.Are you aware of the treatment of the disease? Yes□ No□

9.Are you satisfied with the medical treatment? Very satisfied□ satisfied□ General□ Dissatisfied□ Very dissatisfied□

**The second part: the investigation of expectation and perception** This section is based on the highest standards of 5, to investigate the level of expected medical service quality in the hospital and catual medical services.If you have high expectations for an item, select a higher score, or, on the other hand, select a lower score.In actual feeling part, if you agree with the hospital in a certain content to do better, please choose a higher score; instead, choose a lower score.The investigation content in 1-5 were used to indicate, please according to your actual situation in the most appropriate options on tick“√”.

**a:patients’ expectations**

| Survey content | Expectations | | | | |
| --- | --- | --- | --- | --- | --- |
|  | Not high | Not too high | General | Relatively high | High |
| 1 Modern equipment in the hospital | 1 | 2 | 3 | 4 | 5 |
| 2 Hospital attractiveness | 1 | 2 | 3 | 4 | 5 |
| 3 Hospital medical staff wear clean and decent uniforms | 1 | 2 | 3 | 4 | 5 |
| 4 Attractiveness of medical materials | 1 | 2 | 3 | 4 | 5 |
| 5 Hospitals provide health promotion, service guide and other information | 1 | 2 | 3 | 4 | 5 |
| 6 Providing timely services | 1 | 2 | 3 | 4 | 5 |
| 7 The hospital staff get things done the first time | 1 | 2 | 3 | 4 | 5 |
| 8 The hospital executes your treatment plan with accuracy | 1 | 2 | 3 | 4 | 5 |
| 9 Hospital accurately record your diagnosis and treatment | 1 | 2 | 3 | 4 | 5 |
| 10 The hospital staff communicate to patients about service provision | 1 | 2 | 3 | 4 | 5 |
| 11 The hospital staff provide prompt services | 1 | 2 | 3 | 4 | 5 |
| 12 The hospital pay attention to and deal with your opinions or complaints | 1 | 2 | 3 | 4 | 5 |
| 13 Medical staff willingness to help patients | 1 | 2 | 3 | 4 | 5 |
| 14 The hospital medical staffs are with good medical ethics | 1 | 2 | 3 | 4 | 5 |
| 15 The hospital medical staffs are worth for your trust | 1 | 2 | 3 | 4 | 5 |
| 16 Patient feel safe in the hospital | 1 | 2 | 3 | 4 | 5 |
| 17 Hospital staff are always courteous towards patients | 1 | 2 | 3 | 4 | 5 |
| 18 Medical staff are knowledgeable enough | 1 | 2 | 3 | 4 | 5 |
| 19Knowledgeable personnel to answer patients’ questions | 1 | 2 | 3 | 4 | 5 |
| 20 Medical staff ask you for advice on treatment | 1 | 2 | 3 | 4 | 5 |
| 21 The hospital gives priority to your benefits, not the benefits of medical staff | 1 | 2 | 3 | 4 | 5 |
| 22 The hospital medical expenses are reasonable | 1 | 2 | 3 | 4 | 5 |
| 23 The cost of medical services is issued in a timely and convenient manner | 1 | 2 | 3 | 4 | 5 |
| 24 Detailed list of the items in the hospital charges | 1 | 2 | 3 | 4 | 5 |

**c:patients’ perceptions**

| Survey content | Perceptions | | | | |
| --- | --- | --- | --- | --- | --- |
|  | strongly disagree | disagree | indifferent | agree | strongly agree |
| 1 Modern equipment in the hospital | 1 | 2 | 3 | 4 | 5 |
| 2 Hospital attractiveness | 1 | 2 | 3 | 4 | 5 |
| 3 Hospital medical staff wear clean and decent uniforms | 1 | 2 | 3 | 4 | 5 |
| 4 Attractiveness of medical materials | 1 | 2 | 3 | 4 | 5 |
| 5 Hospitals provide health promotion, service guide and other information | 1 | 2 | 3 | 4 | 5 |
| 6 Providing timely services | 1 | 2 | 3 | 4 | 5 |
| 7 The hospital staff get things done the first time | 1 | 2 | 3 | 4 | 5 |
| 8 The hospital executes your treatment plan with accuracy | 1 | 2 | 3 | 4 | 5 |
| 9 Hospital accurately record your diagnosis and treatment | 1 | 2 | 3 | 4 | 5 |
| 10 The hospital staff communicate to patients about service provision | 1 | 2 | 3 | 4 | 5 |
| 11 The hospital staff provide prompt services | 1 | 2 | 3 | 4 | 5 |
| 12 The hospital pay attention to and deal with your opinions or complaints | 1 | 2 | 3 | 4 | 5 |
| 13 Medical staff willingness to help patients | 1 | 2 | 3 | 4 | 5 |
| 14 The hospital medical staffs are with good medical ethics | 1 | 2 | 3 | 4 | 5 |
| 15 The hospital medical staffs are worth for your trust | 1 | 2 | 3 | 4 | 5 |
| 16 Patient feel safe in the hospital | 1 | 2 | 3 | 4 | 5 |
| 17 Hospital staff are always courteous towards patients | 1 | 2 | 3 | 4 | 5 |
| 18 Medical staff are knowledgeable enough | 1 | 2 | 3 | 4 | 5 |
| 19Knowledgeable personnel to answer patients’ questions | 1 | 2 | 3 | 4 | 5 |
| 20 Medical staff ask you for advice on treatment | 1 | 2 | 3 | 4 | 5 |
| 21 The hospital gives priority to your benefits, not the benefits of medical staff | 1 | 2 | 3 | 4 | 5 |
| 22 The hospital medical expenses are reasonable | 1 | 2 | 3 | 4 | 5 |
| 23 The cost of medical services is issued in a timely and convenient manner | 1 | 2 | 3 | 4 | 5 |
| 24 Detailed list of the items in the hospital charges | 1 | 2 | 3 | 4 | 5 |

**Thank you for your cooperation, I wish you a speedy recovery！**

Investigator：­­­­­­­­­­___________ Survey date：­­­­­____________
